# A case report of a novel inflammatory and incarcerated testicular stromal tumor associated with the *EWSR1::ATF1* gene fusion

**DOI:** 10.1186/s13000-026-01835-2

**Published:** 2026-08-10

**Authors:** Li Wei, Shasha Lv, Qin Guo, Wei Jia

**Affiliations:** 1 Department of Pathology, the First Affiliated Hospital of Shihezi University, Shihezi, Xinjiang 832000 China; 2 Department of Pathology, Hongxing Hospital of the 13th Division of Xinjiang Production and Construction Corps, Xinxing, Xinjiang 839000 China; 3 Department of Pathology, School of Medicine, Shihezi University, Shihezi, Xinjiang 832000 China

**Keywords:** Inflammatory and nested testicular sex cord tumor, EWSR1, ATF1

## Abstract

**Background:**

This case report describes a novel subtype of testicular sex cord-stromal tumor, termed the “inflammatory and nested testicular sex cord tumor”, which is characterized by a specific *EWSR1::ATF1* gene fusion. We present the clinicopathological features of this case to enhance the understanding of this extremely rare tumor.

**Case presentation:**

A 45-year-old male patient presented with a right testicular mass that had been present for six years without an identifiable cause. MRI conducted revealed a round nodule in the right testicular area, measuring approximately 38 × 28 mm, with relatively well-defined margins. The tumor exhibited a nodular growth, consisting of epithelial-like cells with abundant clear cytoplasm arranged in solid nests or sheets. Fibrous connective tissue surrounded the epithelial-like tumor nests, and focal inflammatory cell infiltration was observed within the stroma and tumor periphery. Immunohistochemistry showed membranous positivity for α-inhibin in the epithelial-like component. Molecular testing revealed an *EWSR1::ATF1* gene fusion, leading to a final diagnosis of inflammatory and nested testicular sex cord tumor.

**Conclusions:**

We report a novel subtype of testicular sex cord-stromal tumor, distinct from conventional forms, characterized by nest-like growth of clear epithelial-like tumor cells, focal inflammatory cell infiltration, and the presence of the *EWSR1::ATF1* gene fusion. This case provides further evidence that supports the fusion pattern and clinicopathological basis for the newly proposed subtype of testicular sex cord-stromal tumor, inflammatory and nested testicular sex cord tumor.

## Background

Testicular sex cord-stromal tumors (TSCSTs) are relatively rare, accounting for approximately 4–6% of adult testicular tumors and over 30% of testicular tumors in infants and children [1, 2]. Due to the absence of specific biomarkers, the diagnosis of TSCSTs primarily relies on clinicopathological evaluations. TSCSTs are primarily classified into three subtypes: stromal, Sertoli, and granulosa cell tumors [3]. Lesions that do not conform to these diagnostic categories are generally regarded as “unclassified” TSCSTs. In recent years, immunophenotypic and molecular analyses have deepened the understanding of biological and genomic features of TSCSTs, leading to the inclusion of additional tumor types in the latest WHO classification.

Most TSCSTs present as scrotal masses in either children or adults and are typically confined to the testis at the time of diagnosis [4]. Histologically, these tumors are usually benign and well-differentiated [4]. With advancements in molecular pathology, the detection of specific molecular markers has significantly facilitated the diagnosis and classification of various subtypes. For example, CTNNB1 mutations and β-catenin expression have been confirmed to have diagnostic value in certain Sertoli cell tumors (SCT) [5, 6]. By identifying these molecular features, clinicians can achieve a more accurate diagnosis and classification of tumors and develop individualized treatment strategies.

Recently, inflammatory and nested TSCST (IN-TSCT) has been proposed and defined as a novel subtype of TSCSTs [3]. This tumor is characterized by the presence of an *EWSR1::ATF1* gene fusion, along with distinct histological features, an entrapped growth pattern, and extensive inflammatory cell infiltration [3, 7].

In this study, we report a case of IN-TSCT. Through a comprehensive evaluation of its clinical presentation, pathological features, and molecular characteristics, we further clarified its differences from conventional TSCSTs. Additionally, by comparing the similarities and differences between IN-TSCT and other *EWSR1::ATF1* fusion-associated tumors, this study provides valuable insights into the diagnosis and potential therapeutic strategies for IN-TSCT.

## Case presentation

**Fig. 1:**
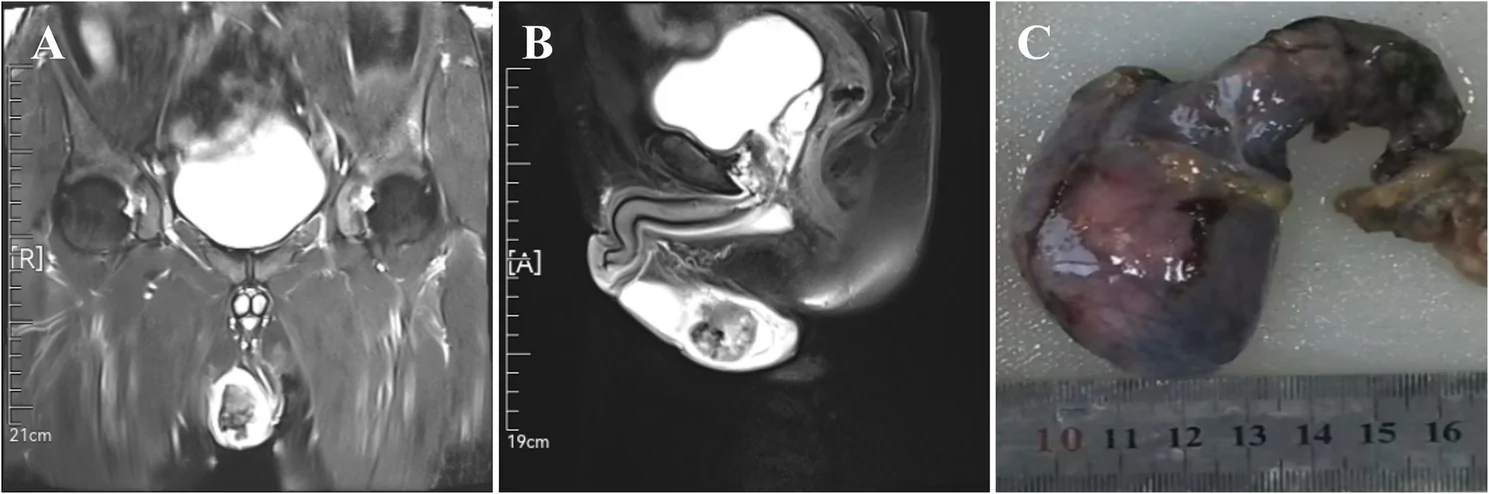
Clinical presentation. **A**, **B** Computed tomography images; (**C**) Testicular gross image

A 45-year-old male patient presented with a right testicular mass that had been present for 6 years without an identifiable cause. Three days before presentation, he experienced mild testicular discomfort, which worsened with physical activity. MRI conducted in January 2024 revealed a round nodule in the right testicular area, measuring approximately 38 × 28 mm, with relatively well-defined margins (Fig. 1A, B). The patient subsequently underwent a high inguinal incision for spermatic cord dissection and complete orchiectomy. The excised testis measured 5 × 4 × 3 cm. Gross examination revealed a greyish-white, round nodule observed on the cut surface, exhibiting a medium texture (Fig. 1C).

**Fig. 2:**
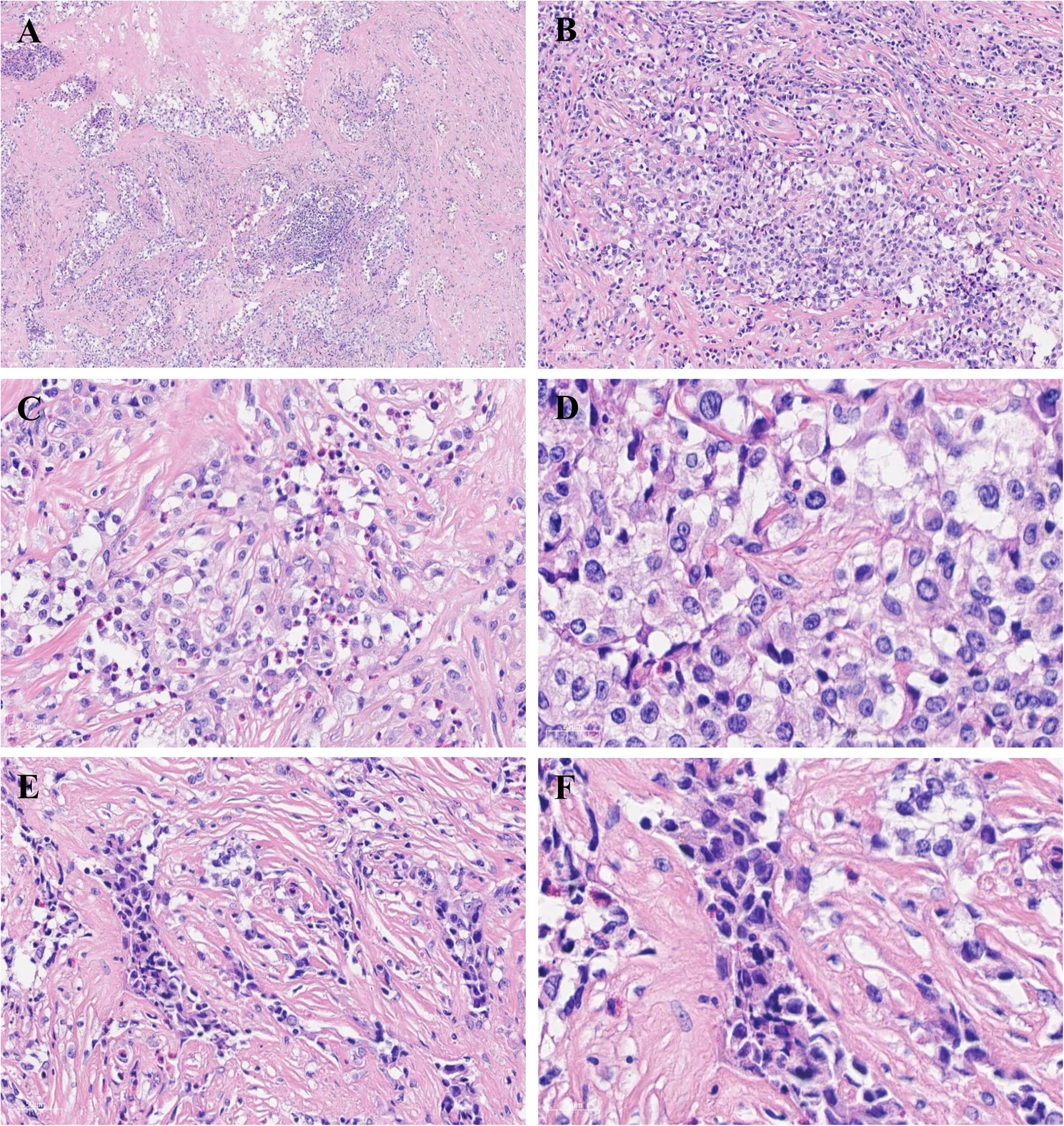
H&E images. **A** Low-power view showing a nodular growth pattern at the tumor margin with focal geographic coagulative necrosis; (**B**) Intermediate-power view showing the tumor arranged in sheet‑like and nested patterns, surrounded by inflammatory cell infiltration; (**C**) Intermediate-power view showing tumor nests encircled by thin fibrous connective tissue infiltrated with inflammatory cells; (**D**) High-power view demonstrating tumor cells with round, centrally located nuclei and clear, spider‑web‑like cytoplasm; (**E**) Intermediate-power view showing tumor cells arranged in slit‑like to cord‑like patterns within sclerotic stroma; (**F**) High-power view demonstrating loss of cytoplasmic clearing, marked nuclear atypia, and discernible pathological mitotic figures

Histopathological examination revealed a nodular tumor mass surrounded by normal testicular tissue, with ill-defined boundaries and irregular infiltration into adjacent structures. No definite lymphovascular invasion was identified. Focal geographic coagulative necrosis was present (Fig. 2A). The tumor was composed of two main components: solid nests or sheets of tumor cells with an epithelial appearance, and scattered aggregates of inflammatory cells (Fig. 2B). The tumor cells exhibited abundant clear cytoplasm, often vacuolated, creating a characteristic web-like pattern. Nuclear atypia was evident, featuring round to oval nuclei with occasional irregular membranes and medium-sized nucleoli. Mitotic figures were readily observed in these areas, averaging 4 per 2 mm². These solid nests of clear cells were surrounded by varying degrees of inflammatory infiltration, primarily by lymphocytes, plasma cells, and eosinophils(Fig. 2C, D). In addition, some tumor cells adopted slit-like or cord-like configurations within a sclerotic stroma. These cells showed loss of cytoplasmic clearing, more pronounced nuclear atypia, and discernible pathological mitotic figures(Fig. 2E, F).

**Fig. 3:**
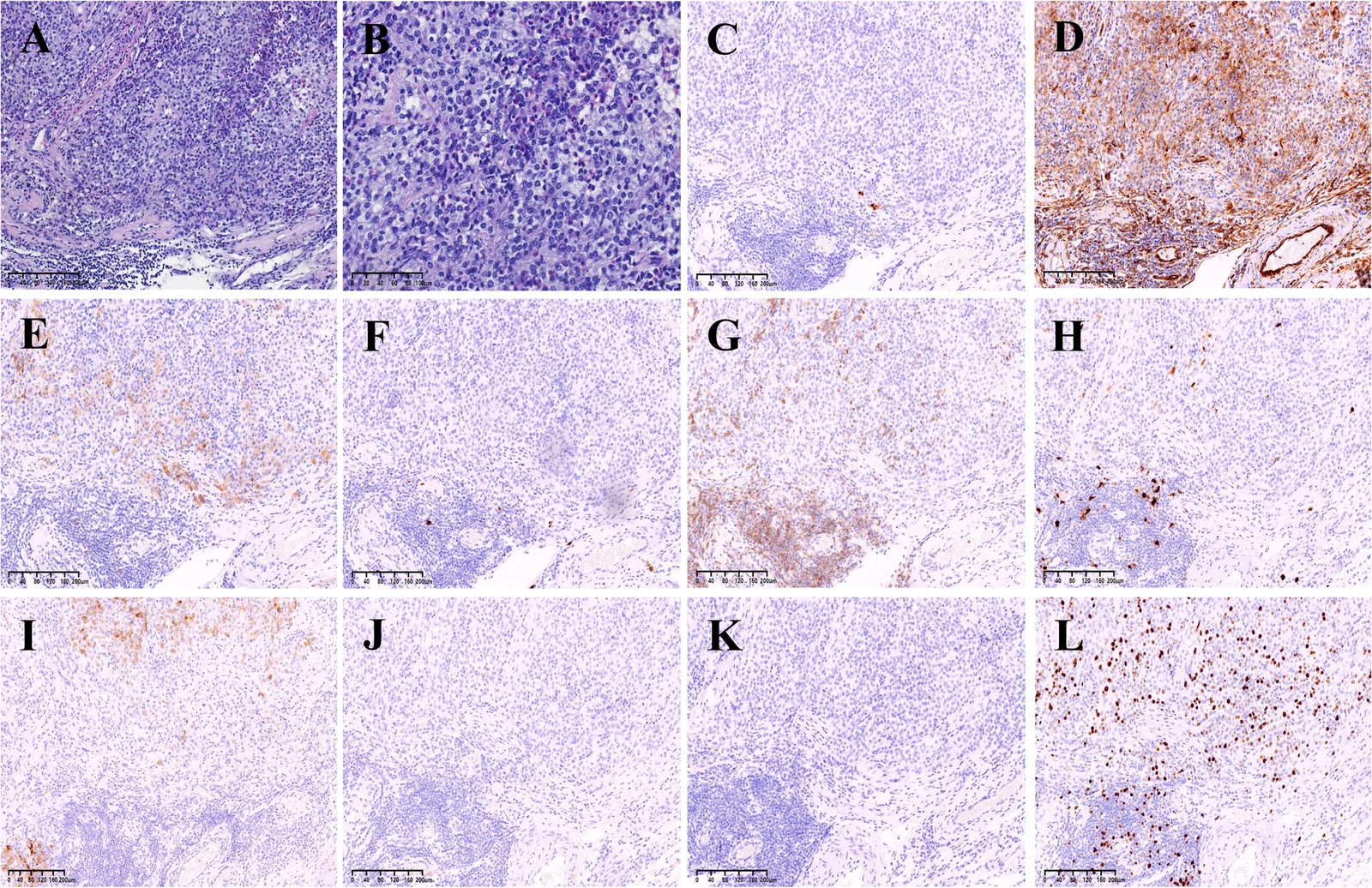
IHC images. **A** Low and intermediate magnification view showing tumor tissue. **B** Intermediate-power view of tumor tissue. **C** Cytokeratin (CK), negative staining. **D** Vimentin, positive staining. **E** CD30, positive staining in peritumoral lymphocytes. **F** CD117, negative staining. **G** Leukocyte common antigen (LCA), positive staining in peritumoral lymphocytes. **H** S100, scattered positive staining. **I** α-inhibin, nest-like positive staining. **J** OCT3/4, negative staining. **K** SALL4, negative staining. **L** Ki-67, nest-like positive staining

Immunohistochemistry showed that the tumor was negative for CK, CD117, OCT3/4, and SALL-4, but positive for vimentin, CD30 (focal), LCA (patchy), S-100 (patchy), α-inhibin (partial), and Ki67 (40%) (Fig. 3).

**Fig. 4:**
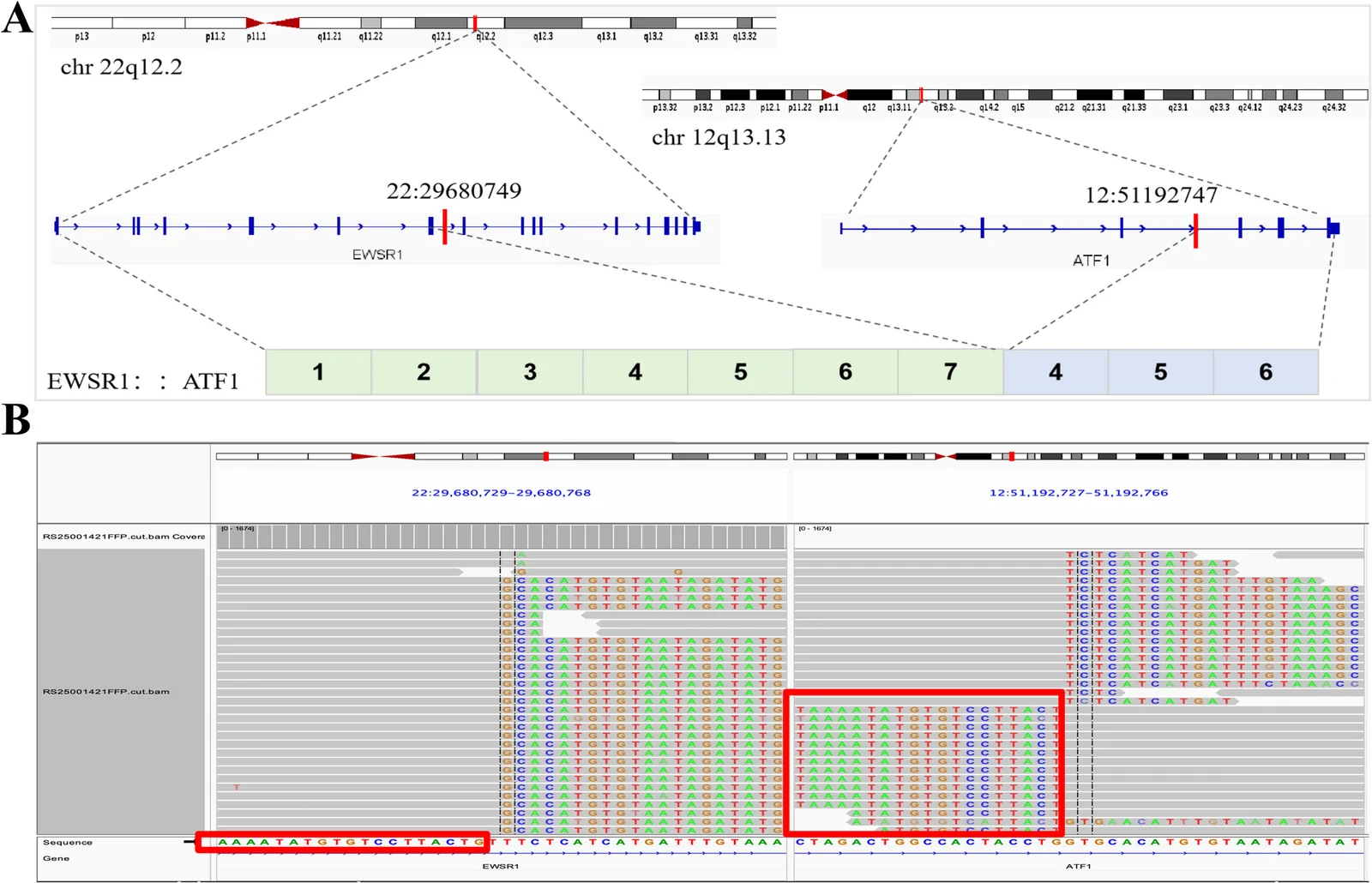
EWSR1::ATF1 gene fusion images. **A** Fusion pattern of the EWSR1::ATF1 gene fusion. **B** Fusion sequence of the EWSR1::ATF1 gene fusion

Histopathological and immunohistochemical findings were insufficient for definitive tumor classification. A review of the literature identified a newly named tumor, inflammatory and entrapped testicular stromal tumor, which exhibited morphological features similar to the tumor in this case. Therefore, next-generation sequencing was performed. Notably, the results revealed that the patient also had an *EWSR1::ATF1* gene fusion (Fig. 4). The final diagnosis was an inflammatory and entrapped testicular stromal tumor with an *EWSR1::ATF1* (22:29680749_12:51192747) gene fusion.

The patient has been followed up for 18 months postoperatively with no evidence of recurrence or metastasis. Surveillance imaging, including testicular MRI and CT scans of the chest and abdomen, has shown only postoperative changes and no signs of metastatic disease to date.

## Discussion

In 2023, Acosta et al. first described a group of testicular stromal tumors with an *EWSR1::ATF1* gene fusion and proposed the designation “inflammatory and nested testicular stromal tumor” for this newly recognized entity [3]. In 2024, Carrillo-Ng et al. reported a case of metastatic IN-TSCT with an *EWSR1::ATF1* gene fusion in the lung [7]. Similarly, in 2025, Yan Wang et al. reported a case of IN-TSCT, which showed no evidence of metastasis on follow-up [8]. In this article, we present a case of an IN-TSCT with *EWSR1::ATF1* gene fusion, further contributing to the newly named tumor entity of IN-TSCT. This case involved a 45-year-old male who presented with a right testicular mass without obvious predisposing factors. The mass was a roundish nodule, and based on histological morphology, immunohistochemistry, and molecular fusion testing, the diagnosis of an IN-TSCT with an *EWSR1::ATF1* gene fusion was made.

Advances in molecular sequencing, an increasing number of SCTs have been found to exhibit unique molecular alterations, continuously enriching the molecular subtypes of SCTs. Recently, aggressive testicular stromal tumors have been categorized into three molecular subgroups: those characterized by FH inactivation, Wnt pathway activation (CTNNB1 or APC mutations), and copy number alterations [9]. SCTs are classified into two main subtypes: not otherwise specified (NOS) and large-cell calcifying SCTs [10, 11]. Approximately 70% of SCTs (NOS) harbour gain-of-function mutations in *CTNNB1*, which are associated with impaired protein degradation and consequent activation of the Wnt signaling pathway [5, 12]. In a cohort of 22 sex cord-stromal NOS/mixed-sex cord-stromal tumors, approximately 30% carried CTNNB1 or APC mutations [13]. Recently, studies have also reported a malignant sex cord tumor with an *EWSR1::ATF1* gene fusion, which is often misdiagnosed as a SCT or seminoma. These tumors are now being categorized as a new independent entity IN-TSCT [3, 7].

IN-TSCT demonstrates distinct histological morphology and immunohistochemical phenotypic characteristics; however, some features still resemble SCT and seminomas, which pose diagnostic challenges [14]. Differentiation relies largely on molecular sequencing results. In the present case, diagnosis was challenging based on tumor location, histology, and immunohistochemistry alone, prompting a differential consideration between SCT and seminoma. SCT primarily occurs in adult males, with an average age of 34 years [15]. It is typically characterized by a unilateral testicular nodule, where tumor cells are arranged in small, solid, or irregular clusters and nests. These tumors are commonly encircled by dense fibrous connective tissue and commonly express Vimentin and α-Inhibin [14, 15]. In contrast, the tumor in our case was composed of nest-like epithelial cells encircled by fibrous connective tissue, with extensive inflammatory cell infiltration. In addition to the predominant nested growth pattern, focal areas exhibited a slit-like or cleft-like arrangement of tumor cells; these areas were somewhat more prominent adjacent to necrotic foci. Compared with the epithelioid cells within nests, those in the slit-like areas had scantier, less clear cytoplasm, more pronounced nuclear atypia, and a higher mitotic rate. Furthermore, small necrotic foci were present within these slit-like areas, along with locally deposited hemosiderin. We hypothesize that the slit-like growth pattern may be associated with tumor necrosis.

Seminoma, the most common type of testicular germ cell tumor, are typically arranged in sheets, nests, and cords, with fibrous septa around the nests. Tumor cells often have a clear cytoplasm, and lymphocytic infiltration can be observed in the stroma. However, seminomas usually express CD117, OCT3/4, and SALL4, which were not observed in our case [4]. This case exhibited both nest-like clear cells and focal lymphocytic infiltration. In combination with the immunohistochemical positivity for Vimentin and α-Inhibin, it was more likely to be a special type of SCT. High-throughput sequencing revealed an *EWSR1::ATF1* (22:29680749_12:51192747) gene fusion, which confirmed the diagnosis of IN-TSCT. The diagnosis of IN-TSCT requires a comprehensive integration of histological morphology, immunohistochemistry, and molecular characteristics to establish a clear diagnosis. Our case report supports the reproducibility of the *EWSR1::ATF1* fusion in the newly named tumor entity, IN-TSCT.

The EWSR1 gene, located on the human chromosome 22q12.2, encodes an RNA-binding protein consisting of 17 exons and 16 introns [16, 17]. These abnormalities are closely associated with various soft tissue sarcomas and bone tumors [17]. The N-terminal domain of EWSR1 is rich in low-complexity regions and fuses with the C-terminal DNA-binding domains of several transcription factors, such as FLI1, ATF1, CREB1, and WT1, to form chimeric proteins [18]. The ATF1 gene is located on chromosome 12 at 12q13.13 and consists of seven exons and six introns [19]. It activates the target gene expression by binding to the cAMP response element, which participates in oxidative stress and DNA damage repair [20]. Various rearrangement patterns exist in *EWSR1::ATF1* chromosomal fusions. For example, in clear cell sarcomas, the 8th exon of EWSR1 fuses with the 4th exon of ATF1 [21]. In salivary gland clear cell carcinoma, the 11th exon of EWSR1 fuses with the 3rd exon of ATF1 [22]. Similarly, in clear cell sarcomas, in-frame fusion occurs between the 7th exon of EWSR1 and the 7th exon of ATF1 [23]. In this case, the fusion between the 7th intron of EWSR1 and the 3rd intron of ATF1 further supplements the fusion pattern of EWSR1 and ATF1 in IN-TSCT.

While the prognosis for most testicular sex cord-stromal tumors (TSCSTs) is generally favorable, with only approximately 10% exhibiting malignant behavior at presentation or during follow-up, the emerging entity of IN-TSCT appears to diverge from this pattern based on limited reported cases. In the 11 cases of IN - TSCT first reported by Acosta et al., a subset of patients developed metastases to retroperitoneal lymph nodes, non - retroperitoneal lymph nodes, and/or visceral sites (contralateral testis, lung/pleura, mediastinum, and bone) [3]. Additionally, a patient reported by Carrillo - Ng et al. was found to have lung metastases at the time of diagnosis [7]. In contrast, a patient in the report by Wang et al. remained free of metastasis after 10 months of follow-up [8]. In our case, the patient has been followed for 18 months postoperatively without evidence of recurrence or metastasis.

As summarized in Table 1, reported cases of IN-TSCT demonstrate heterogeneous clinical features and outcomes; while metastases have been documented in several instances, other patients, including the present case, have remained free of metastasis during follow-up. Due to the limited number of cases and relatively short follow-up periods, the long-term clinical behavior and potential aggressiveness of IN-TSCT remain unclear, and there is currently no definitive evidence to classify it as a consistently aggressive tumor. This study has several limitations that reflect this broader uncertainty: it is a single-case report, and the follow-up duration is insufficient to assess long-term outcomes. Therefore, whether IN-TSCT is more aggressive than conventional sex cord-stromal tumors requires validation through larger, multi-center studies with longer follow-up.

**Table 1:** Comparison between previously reported cases and the present case

	Age	Tumor Size(cm)	Histopathologic features	NGS results	FISH	Treatment	Follow up (months)	Metastasis	Status
Acosta et al.	25	NA	S + N	22:29682866_ 12:51199238	EWSR1::ATF1	S + C+*R*	96	lung	A
	39	NA	S + N+G	22:29678636_ 12:51197939	EWSR1::ATF1	S + C	60	Vertebral	A
	20	2	S + G	NP	EWSR1::ATF1	S + R	19	Retroperitoneal	A
	29	4	S + N+G	NP	EWSR1::ATF1	S	35	Axillary LN and lung	A
	37	2.5	S + N+G	NP	EWSR1::ATF1	S + R	38	Retroperitoneal	A
	29	NA	S + N+G	NP	EWSR1::ATF1	S	11	Retroperitoneal LN and contralateral testis	A
	38	0.9	S + N	NP	EWSR1::ATF1	S	NA	NA	NA
	36	2.5	S + N+G	NP	EWSR1::ATF1	S + R	40	Retroperitoneal LN and left supraclavicular LN	A
	31	3	S + N+G	NP	EWSR1::ATF1	S + R	14	Retroperitoneal LN and lung	D
	60	5.5	S + G	NP	Negative	S	4	Pleural effusion	A
	33	3.5	S + N+G	22:29678365_ 12:51198449	EWSR1::ATF1	S	27	Lung and mediastinum	D
	48	3	S + G	NP	ATF1 amplification	S	NA	NA	NA
	73	4.5	S + N+G	NP	Negative	S	NA	NA	NA
Carrillo-Ng et al.	54	NA	S + N+G	EWSR1::ATF1, TP53 frameshift	NP	NA	2	Lung	D
Yan Wang et al.	36	6.0	S + N+G	NP	EWSR1 Break	S + C	10	N	A
Present case	45	3.8	S + N+G	22:29680749_ 12:51192747	NP	S	18	N	A

*NA* not available, Histopathologic features (*S* solid sheets, *N* nests, *G *geographic necrosis), *NP* not performed, Treatment (*S* surgical excision, *C* chemotherapy, *R* radiotherapy), Metastasis (*M* metastasis, *N* no evidence of metastasis), Recurrence (*R* recurrence, *N* no evidence of recurrence), Status (*A* Alive, *D* Dead)

## Conclusions

We report a case of IN-TSCT harbouring an EWSR1:ATF1 gene fusion. This case further provides evidence to support the classification of IN - TSCT as a distinct entity characterized by specific morphological and immunological features, along with recurrent *EWSR1::ATF1* fusions.

## Data Availability

Not applicable. Our manuscript does not contain any numerical data.

## Abbreviations

IN-TSCT: Inflammatory and nested testicular sex cord-stromal tumor
NOS: Not otherwise specified
SCT: Sertoli cell tumor
TSCST: Testicular sex cord-stromal tumor
